# Hybridization Properties of RNA Containing 8-Methoxyguanosine and 8-Benzyloxyguanosine

**DOI:** 10.1371/journal.pone.0137674

**Published:** 2015-09-09

**Authors:** Daniel Sylwester Baranowski, Weronika Kotkowiak, Ryszard Kierzek, Anna Pasternak

**Affiliations:** Institute of Bioorganic Chemistry, Polish Academy of Sciences, Noskowskiego 12/14, 61-704, Poznan, Poland; University of Quebec at Trois-Rivieres, CANADA

## Abstract

Modified nucleobase analogues can serve as powerful tools for changing physicochemical and biological properties of DNA or RNA. Guanosine derivatives containing bulky substituents at 8 position are known to adopt *syn* conformation of N-glycoside bond. On the contrary, in RNA the *anti* conformation is predominant in Watson-Crick base pairing. In this paper two 8-substituted guanosine derivatives, 8-methoxyguanosine and 8-benzyloxyguanosine, were synthesized and incorporated into oligoribonucleotides to investigate their influence on the thermodynamic stability of RNA duplexes. The methoxy and benzyloxy substituents are electron-donating groups, decreasing the rate of depurination in the monomers, as confirmed by N-glycoside bond stability assessments. Thermodynamic stability studies indicated that substitution of guanosine by 8-methoxy- or 8-benzyloxyguanosine significantly decreased the thermodynamic stability of RNA duplexes. Moreover, the presence of 8-substituted guanosine derivatives decreased mismatch discrimination. Circular dichroism spectra of modified RNA duplexes exhibited patterns typical for A-RNA geometry.

## Introduction

Chemically modified nucleic acids are a quickly growing class of compounds that have various uses. They can be successfully applied in investigations of nucleic acid structure and interactions between nucleic acids and proteins. Nucleoside analogs also exhibit promising properties *in vitro* and *in vivo* as therapeutic agents, e.g., in gene silencing or in anti-viral and cancer therapy. In biological studies, they often serve as enzyme inhibitors and are able to inactivate the replication, transcription, translation, repair and processing pathways of nucleic acids [1–3].

More than two decades ago, research began to focus on purine nucleosides containing various modifications at the 8-position [4,5]. The presence of bulky substituents at the 8-position of a purine moiety shifts the equilibrium of the N-glycoside bond towards the *syn* conformation. The 8-substituted purine nucleosides exhibit promising anti-proliferative properties by inducing apoptosis of different cancer cell lines, *e*.*g*., multiple myeloma and other leukemias [5–9]. Moreover, purine nucleosides modified at position C-8 are promising bacterial inhibitors and offer an interesting alternative to currently known antibiotics [10,11]. 8-substituted purine 2’-deoxyribosides have been applied to probe the conformational preferences of the N-glycoside bonds within DNA duplexes and quadruplexes [12,13]. The incorporation of 8-substituted purine nucleosides into DNA has been shown to stabilize the Z-DNA form [14,15].

RNA consists of canonical and non-canonical secondary structure motifs, such as internal loops, hairpins, and bulges. One of the most ubiquitous types of RNA secondary structure motifs are single mismatches [16]. All these motifs are biologically relevant for providing binding sites for proteins, small molecules, or nucleic acids [16–20]. Chemically modified nucleotides can either increase or decrease mismatch discrimination (increase or decrease base pairing specificity). The modifications that increase the hybridization specificity *i*.*e*. increase thermodynamic stability of duplexes are promising tools in allele-specific PCR, antisense therapies or in the detection of single nucleotide polymorphisms (SNPs) [21]. On the other hand, modified nucleotides that decrease base pairing specificity *i*.*e*. decrease thermodynamic stability of duplexes allow to use of primers and probes when it is required to detect hybridization of multiple sequences simultaneously (hybridization independent of base composition) and minimize mismatch discrimination [21]. These modifications can also be successfully applied as molecular tools to improve siRNA-mediated gene silencing by reducing “off target” effect [22].

Herein, we report the chemical synthesis of 8-methoxyguanosine (mxG) and 8-benzyloxyguanosine (bxG) phosphoramidites and their incorporation into RNA using standard β-cyanoethyl chemistry and automated RNA synthesis. The hybridization properties of modified oligoribonucleotides were thermodynamically characterized by UV melting method, and the influence of complementary mxG-C and bxG-C base pairs, as well as of mxG and bxG mismatches with A, G and U, was determined. Moreover, RNA duplexes containing 8-substituted derivatives of guanosine were analyzed by circular dichroism to investigate possible alterations in helix geometry. In addition, the acidic stability of the N-glycoside bond of 8-methoxyguanosine and 8-benzyloxyguanosine was determined since the formation of abasic sites changes the RNA secondary structure.

## Materials and Methods

### Chemical synthesis of 8-methoxyguanosine and 8-benzyloxyguanosine phosphoramidites

All details concerning chemical synthesis of 8-substituted guanosine derivatives are described in S1 File.

### Oligonucleotides

All oligonucleotides were synthesized on an automated RNA/DNA synthesizer using standard phosphoramidite chemistry [23]. The 8-substituted guanosine phosphoramidite monomers were applied according to previously described procedures together with commercial DNA and RNA phosphoramidites. Details of the deprotection and purification of oligoribonucleotides have been previously described [24,25]. The composition of all oligonucleotides was confirmed using MALDI-TOF mass spectrometry.

### N-glycoside bond stability studies

The rate of depurination process was tested for both 8-substituted guanosine derivatives and compared with the stability of guanosine. To the 30 μl of 14 mM solution of corresponding nucleoside, 240 μl of water and 30 μl of 1M HCl were added. The surface of reaction mixture was tightly covered by silicone oil and heated at 85°C. Aliquots of reaction mixture were removed after 0, 0.25, 0.5, 1, 2, 4, 6, 8, and 24 hours and the oil left-overs were removed. The hydrolysis reaction was quenched each time by addition of the excess of 30% aqueous ammonia. The relative amount of the substrate and product was determined by HPLC with UV detection at 220-300nm wavelength range.

### UV-vis melting analysis

Oligonucleotides were dissolved in a buffer containing 100 mM sodium chloride, 20 mM sodium cacodylate and 0.5 mM Na_2_EDTA, pH 7.0. Single-stranded oligonucleotide concentrations were calculated based on the absorbance measured above 80°C and extinction coefficients were approximated using the Oligo Calculator from RiboTask ApS website (www.ribotask.com). Modified and unmodified RNA strands with identical sequences were assumed to have identical extinction coefficients. The samples were denatured at 90°C for 2 min and then cooled to room temperature. The measurements were performed for nine different concentrations of each sample in the concentration range of 10^−4^–10^-6^ M. Melting curves were obtained using the UV melting method at 260 nm in the temperature range of 3–93°C with a heating rate of 1°C/min on a JASCO V-650 spectrophotometer equipped with a thermoprogrammer. Melting curves were analyzed and the thermodynamic parameters determined by nonlinear curve fitting using the MeltWin 3.5 software [26]. The ΔH° derived from T_m_^-1^ versus ln (C_T_/4) plots was within 15% of that derived from averaging the fits to individual melting curves for majority of analysed duplexes (S1 Fig and S1 Table). This indicates that the two-state model is valid for most duplexes studied herein. Melting temperatures calculated for a 10^-4^ M concentration of oligonucleotide are denoted by T_M_, and melting points for any other concentration of oligonucleotide are denoted by T_m_.

### CD spectroscopy

CD spectra were recorded on a JASCO J-815 spectropolarimeter. The oligonucleotides were dissolved in the same buffer as for UV melting studies to achieve a sample concentration of 4.9 μM. All samples were denatured at 90°C for 2 min and then cooled to room temperature prior to data collection. The spectra were recorded in triplicate at 5°C in the 205–320 nm wavelength range with a 1 nm data interval. The buffer spectrum was subtracted from the sample spectra.

## Results and Discussion

### Thermodynamics of RNA duplexes containing 8-methoxyguanosine and 8-benzyloxyguanosine

An 8-methoxy or 8-benzyloxy group was expected to significantly destabilize RNA duplexes. The bulky substituents at the 8-position preferentially shift the N-glycoside bond of guanosine towards the *syn* conformation, whereas natural Watson-Crick base pairs within RNA duplexes require an *anti-*conformation to be formed. Thus, the hybridization process is energetically costly due to the presence of steric bulk and the need for accommodation by mxG or bxG, the *anti*-conformation within an RNA duplex.

8-methoxyguanosine was incorporated into the central position of a 9 nt oligoribonucleotide. The presence of mxG decreases RNA duplex stability by 1.87 kcal/mol (Table 1), which corresponds to a 20-fold decrease of the binding constant. The destabilization was comparable but slightly lower relative to the destabilizing effect observed for the incorporation of 8-methoxy-2′-deoxyriboadenosine within the DNA duplex (ΔΔG°_37_ = 2.40 kcal/mol) [12] or 8-bromoguanosine within the stem of the RNA hairpin (ΔΔG°_37_ = 2.36 kcal/mol) [27].

**Table 1:** Thermodynamic parameters of duplex formation.^a^

Duplexes (5'-3')		T_M_^-1^ vs log C_T_ plots							
		-ΔH° (kcal/mol)	-ΔS°(eu)	-ΔG°_37_ (kcal/mol)	T_M_ ^b^ (°C)	ΔΔG°_37_ (kcal/mol)	ΔT_M_ ^b^ (°C)	ΔΔG°_37_ (kcal/mol)	ΔT_M_ ^b^ (°C)
GAUCGACAG	CUGUCGAUC	80.0±1.3	222.4±4.1	10.98±0.05	55.3	0	0	-	-
GAUC**G**^**Bx**^ACAG	CUGUCGAUC	(100.1±5.4)	(299.4±17.4)	(7.29±0.05)	(39.3)	3.69	-16.0	**0**	**0**
GAUC**G**^**Mx**^ACAG	CUGUCGAUC	72.7±2.8	204.9±8.7	9.11±0.07	48.4	1.87	-6.9	*0*	*0*
GAUCGACAG	CUGUAGAUC	57.7±4.3	166.7±14.2	6.03±0.12	34.3	4.95	-21.0	-	-
GAUC**G**^**Bx**^ACAG	CUGUAGAUC	(75.5±4.2)	(217.6±13.4)	(7.99±0.05)	(43.1)	-	-	**-0.70**	**3.8**
GAUC**G**^**Mx**^ACAG	CUGUAGAUC	53.4±3.4	153.6±11.2	5.71±0.13	32.3	-	-	*3*.*40*	*-16*.*1*
GAUCGACAG	CUGUGGAUC	66.0±2.5	190.7±8.2	6.80±0.04	38.3	4.18	-17.0	-	-
GAUC**G**^**Bx**^ACAG	CUGUGGAUC	(146.0±7.7)	(452.6±25.1)	(5.66±0.10)	(35.2)	-	-	**1.63**	**-4.1**
GAUC**G**^**Mx**^ACAG	CUGUGGAUC	79.3±1.5	230.8±4.7	7.67±0.01	41.5	-	-	*1*.*44*	*-6*.*9*
GAUCGACAG	CUGUUGAUC	77.1±1.5	222.9±4.8	7.91±0.01	42.6	3.07	-12.7	-	-
GAUC**G**^**Bx**^ACAG	CUGUUGAUC	(102.6±6.1)	(315.8±20.2)	(4.68±0.20)	(31.5)	-	-	**2.61**	**-7.8**
GAUC**G**^**Mx**^ACAG	CUGUUGAUC	75.6±1.2	223.1±4.0	6.37±0.02	36.4	-	-	*2*.*74*	*-12*.*0*

**G**^**Mx**^– 8-methoxyguanosine, **G**^**Bx**^– 8-bezyloxyguanosine

^a^ 100 mM NaCl, 20mM sodium cacodylate, 0.5 mM Na_2_EDTA, pH 7.0

^b^—calculated for 10^−4^ M oligomer concentration

The presence of a methoxy group at the 8-position of guanosine decreases mismatch discrimination (Table 1). The largest destabilization, up to 3.40 kcal/mol, was observed for mxG-A relative to complementary RNA duplex containing an mxG-C base pair (Table 1). mxG-U was less destabilizing (ΔΔG°_37_ = 2.74 kcal/mol), whereas the presence of an mxG-G mismatch decreased the thermodynamic stability of duplexes by 1.44 kcal/mol compared with complementary modified RNA duplex. In contrast, natural mismatches destabilized RNA duplex by 4.95 kcal/mol (G-A), 3.07 kcal/mol (G-U), and 4.18 kcal/mol (G-G). The decrease of binding specificity of RNA duplexes containing 8-methoxyguanosine compared with mismatched unmodified RNA duplexes is most likely the result of prior local distortion of helix due to the presence of a bulky modification. Hence, the presence of mismatches within modified RNA duplex causes slighter energetic loss relative to unmodified duplex.

The melting behavior of RNA duplexes modified with 8-benzyloxyguanosine (bxG) was clearly non-two state. The large discrepancy between thermodynamic parameters derived from T_m_^-1^ versus ln (C_T_/4) plots and averaging the fits to individual melting curves prevented obtaining reliable thermodynamic data for those duplexes (S1 Table). In general, the presence of 8-benzyloxyguanosine at the central position of oligoribonucleotides appears to destabilize RNA duplexes more strongly than 8-methoxyguanosine (Table 1). The decrease of melting temperature of RNA duplex caused by the benzyloxy group at position 8 of guanosine reaches 16°C, what simultaneously makes T_M_ of the duplex more unfavorable by 9.1°C relative to the RNA modified by 8-methoxyguanosine. Additionally, the presence of 8-benzyloxyguanosine decreased mismatch discrimination by 5–25°C relative to unmodified mismatches of the same type. Surprisingly, bxG-A mismatch appeared to increase duplex stability by nearly 4°C relative to RNA duplex with complementary bxG-C interactions. The benzyloxy group is more bulky than the methoxy group. The non-two state behavior is likely the result of a strong local disruption of the helix in its central part. Thus, dissociation of the strands occurs via intermediate forms.

### Circular dichroism spectra

**Fig 1:**
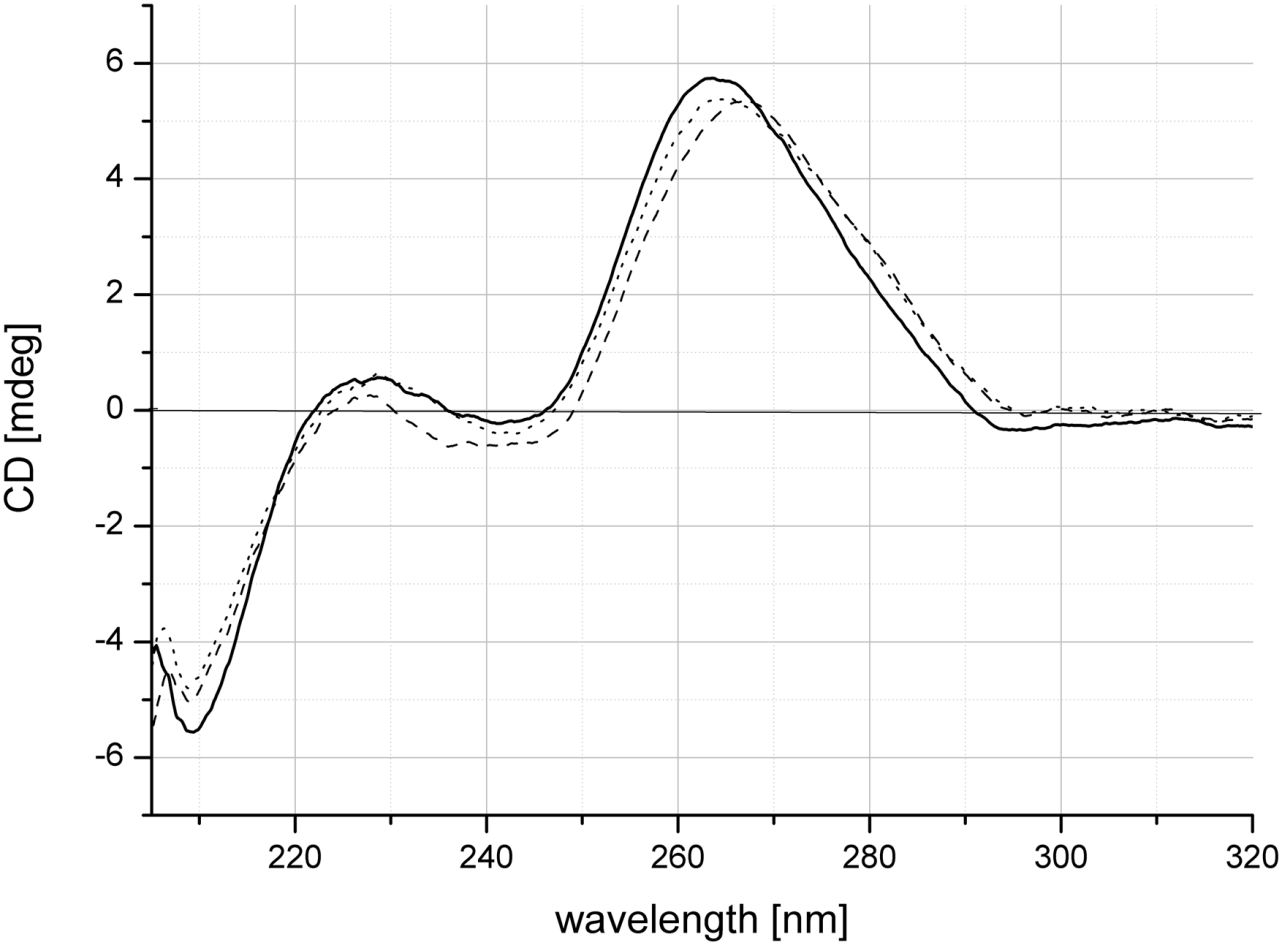
Circular dichroism spectra of unmodified RNA duplexes (solid line) and RNA duplexes modified with 8-methoxyguanosine (dotted line) and 8-benzyloxyguanosine (dashed line).

CD spectra were performed for unmodified RNA duplex and two modified duplexes containing mxG-C and bxG-C base pairs in the central part of the duplex to examine possible alterations in helix geometry (Fig 1). All recorded spectra in the 205–320 nm range were characterized by a positive intense band near ~260 nm and two negative bands at ~210 nm and ~240 nm. Such signals are typical for A-type helix geometry assigned to RNA duplexes. The nearly identical patterns of the CD spectra indicated that both 8-substituted guanosines did not alter the substance of the overall helical geometry [28].

### N-glycoside bond stability studies

**Fig 2:**
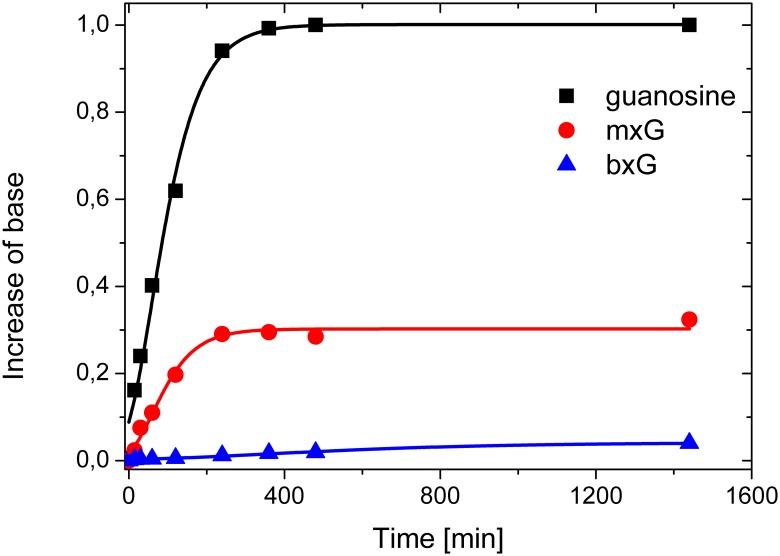
Depurination process as a function of time for guanosine (black, square), 8-methoxyguanosine (red circles) and 8-benzyloxyguanosine (blue triangles).

Depurination is an important process in the chemistry of nucleic acids *in vitro* and *in vivo* [29,30]. Glycosidic hydrolysis results in the formation of abasic sites and thus changes the RNA or DNA secondary structure. The presence of apurinic sites is also responsible for mutations during enzymatic synthesis of nucleic acids *in vivo*. Moreover, depurinated by mutagenic compounds DNA has been suggested to play a key role in cancer initiation. 8-substituted nucleosides are usually chemically stable during oligonucleotide synthesis. However, there are reports concerning some synthetic problems with oligonucleotides modified *i*.*e*., with 8-bromoadenosine [12]. Studies of N-glycoside bond stability have been performed in acidic conditions for guanosine, 8-methoxyguanosine and 8-benzyloxyguanosine at 85°C. HPLC analysis of guanosine increases have indicated that both compounds undergo acidic hydrolysis less rapidly than guanosine (Fig 2). After 85 minutes of the reaction, half of the initial amount of guanosine was depurinated, whereas the remaining two modified nucleosides did not release 50% of the free base, even after 24 hours. However, our data indicate that the order of N-nucleoside bond stability can be classified as bxG > mxG > G. These results are in accordance with Laayoun et al. [4], who have found that the electron-withdrawing character of the substituents at the 8 position of 2′-deoxyadenosine or 2′-deoxyguanosine strongly corresponds with the increased hydrolysis rate of the N-glycosidic bond. Similarly, in our studies, the inductive contribution of the substituent was observed. Methoxy and benzyloxy groups exhibited an electron-donating character, which is reflected in the decreased rate of hydrolysis.

## Conclusions

The results presented herein confirm that a methoxy or benzyloxy group introduced at the 8 position of a guanosine moiety significantly increases the stability of the N-glycoside bond. At the best of our knowledge, this thermodynamic analysis is the first performed for RNA duplexes modified with 8-methoxy- or 8-benzyloxyguanosine. RNA duplexes containing 8-methoxy- or 8-benzyloxyguanosine are less stable compared with unmodified RNA due to the steric bulk of the substituents. Such modified RNA duplexes are also characterized by reduced hybridization specificity due to decreased discrimination of mismatches formed between mxG or bxG with A, G or U. However, the CD curves indicate that the presence of such bulky substituents at the 8 position of a guanosine moiety does not influence A-type geometry, which is characteristic of the RNA helix. As a consequence, oligonucleotides modified with 8-methoxy- and 8-benzyloxyguanosine might be useful tools to simultaneously detect hybridization of multiple sequences or to reverse the relative thermodynamic stability of siRNA ends, thus minimizing off target effects and increasing the rates of gene silencing [22,31]. Moreover, mxG and bxG as a nucleotide analogs with an enforced *syn* conformation, presumably could serve as convenient modifications to increase ribozymes activity or improve antiproliferative properties of G-quadruplex based aptamers targeted towards cancer cells [32,33]. Nevertheless further systematic studies on oligonucleotides modified with mxG and bxG are needed.

## Supporting Information

S1 Fig Representative MeltWin 3.5 software fittings for RNA duplex modified with 8-methoxyguanosine (A) and 8-benzyloxyguanosine (B). Thermodynamic parameters are calculated according to two independent methods. The first method is a van't Hoff analysis of the data by assuming a two-state model. The absorbance versus temperature profile is used to determine the temperature dependence of the equilibrium constant, allowing the calculation of ΔH° and ΔS° for the transition from a van't Hoff plot. The concentration dependence of the T_M_ provides the second van't Hoff method for calculating folding thermodynamics. Moreover, the dependence of the T_M_ on the oligonucleotide strand concentration reveals the molecularity of the transition. More details can be found in “Spectrophotometry and Spectrofluorimetry, Practical approach” edited by Michael G. Gore, chapter 13, p. 329, “The use of spectroscopic techniques in the study of DNA stability” authored by John SantaLucia Jr.
(DOCX)

S1 File Chemical synthesis of 8-methoxyguanosine and 8-benzyloxyguanosine phosphoramidites. (DOCX)

S1 Table Thermodynamic parameters of duplex formation. (DOCX)
