# Supplementary material for: Hybridization Properties of RNA Containing 8-Methoxyguanosine and 8-Benzyloxyguanosine
Source: PLoS One. 2015 Sep 9;10(9):e0137674. doi: 10.1371/journal.pone.0137674 (PMC4564172; doi:10.1371/journal.pone.0137674)
Supplement: S1 Fig — for RNA duplex modified with 8-methoxyguanosine (A) and 8-benzyloxyguanosine (B). Thermodynamic parameters are calculated according to two independent methods. The first method is a van't Hoff analysis of the data by assuming a two-state model. The absorbance versus temperature profile is used to determine the temperature dependence of the equilibrium constant, allowing the calculation of ΔH° and ΔS° for the transition from a van't Hoff plot. The concentration dependence of the TM provides the second van't Hoff method for calculating folding thermodynamics. Moreover, the dependence of the TM on the oligonucleotide strand concentration reveals the molecularity of the transition. More details can be found in “Spectrophotometry and Spectrofluorimetry, Practical approach” edited by Michael G. Gore, chapter 13, p. 329, “The use of spectroscopic techniques in the study of DNA stability” authored by John SantaLucia Jr. (DOCX) [file pone.0137674.s001.docx]

A.


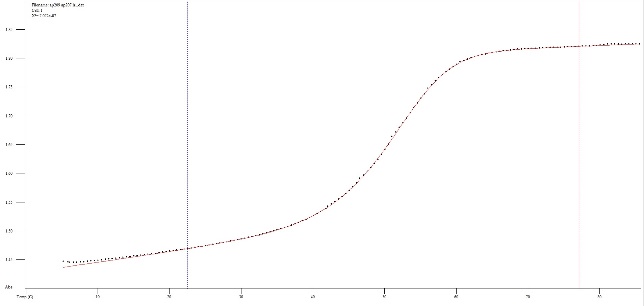

B.


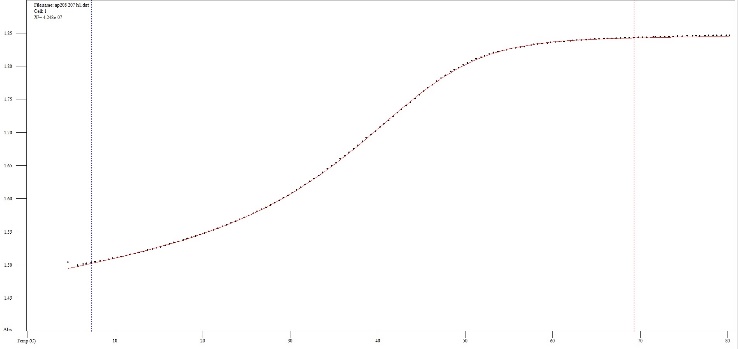

**S1 Fig. Representative MeltWin 3.5 software fittings** for RNA duplex modified with 8-methoxyguanosine (A) and 8-benzyloxyguanosine (B). Thermodynamic parameters are calculated according to two independent methods. The first method is a van't Hoff analysis of the data by assuming a two-state model. The absorbance versus temperature profile is used to determine the temperature dependence of the equilibrium constant, allowing the calculation of ΔH° and ΔS° for the transition from a van't Hoff plot. The concentration dependence of the T_M_ provides the second van't Hoff method for calculating folding thermodynamics. Moreover, the dependence of the T_M_ on the oligonucleotide strand concentration reveals the molecularity of the transition. More details can be found in “Spectrophotometry and Spectrofluorimetry, Practical approach” edited by Michael G. Gore, chapter 13, p. 329, “The use of spectroscopic techniques in the study of DNA stability” authored by John SantaLucia Jr.
