# Supplementary material for: Hybridization Properties of RNA Containing 8-Methoxyguanosine and 8-Benzyloxyguanosine
Source: PLoS One. 2015 Sep 9;10(9):e0137674. doi: 10.1371/journal.pone.0137674 (PMC4564172; doi:10.1371/journal.pone.0137674)
Supplement: S1 File — (DOCX) [file pone.0137674.s002.docx]

**S1 File. Chemical synthesis of 8-methoxyguanosine and 8-benzyloxyguanosine phosphoramidites**

**8-Bromoguanosine**

Bromine water (27 mmol in 170 ml) was added portionwise to a suspension of guanosine (4.0 g, 14.1 mmol) in 60 ml of water and then mixture was stirred for 3h at room temperature. The precipitate was filtered and crystallized from water and dried over P_2_O_5_ in vacuo to yield 4.8 g (93%).

NMR (500 MHz, DMSO-d_6_) δ (ppm) ^1^H NMR 10.82 (brs, 1H, N1-H), 6.51 (brs, 2H, NH_2_), 5.68 (d, 1H, H1’), 5.46, 5.10 (d, d, 1H, 1H, 2’-OH, 3’OH), 5.01 (dd, 1H, H2’), 4.92 (1H, t, 5’OH), 4.15-4.11 (1H, m, H3’), 3.87-3.82 (1H, m, H4’), 3.68-3.61 (1H, m, H5’), 3.54-3.46 (1H, m, H5’’).

**8-Methoxyguanosine**

Solution of 0.80 g of sodium dissolved in 30 ml of anhydrous methanol was added to a solution of bromoguanosine (3.62 g, 10mmol) in 60ml of DMSO. The reaction mixture was heated at 65°C in an oil bath for 20h. The solution was cooled down to room temperature, neutralized with glacial acetic acid, diluted with acetone (100 ml) and poured into 450 ml of diethyl ether. The resulting precipitate was filtered, washed with acetone followed by cold water and then purified by crystallization from water and dried over P_2_O_5_ in vacuo to yield 1.96 g (62%).

NMR (500 MHz, DMSO-d_6_) δ (ppm) ^1^H 10.56 (brs, 1H, N1-H), 6.31 (brs, 2H, NH_2_), 5.59 (d, ^3^*J*_HH_=6.2 Hz, 1H, H1’), 5.31 (d, ^3^*J*_HH_=6.1 Hz, 1H, 2’OH), 5.00 (d, ^3^*J*_HH_=5.1 Hz, 1H, 3’OH), 4.84 (t, ^3^*J*_HH_=5.8, 6.2 Hz, 1H, 5’OH), 4.72 (q, ^3^*J*_HH_=5.9, 5.7, 6.0 Hz, 1H, H2’), 4.04 (q, ^3^*J*_HH_=5.0, 3.7, 5.1 Hz, 1H, H3’), 3.96 (s, 3H, OCH_3_), 3.80-3.77 (m, 1H, H4’), 3.59-3.53 (dt, ^2^*J*_HH_=11.6 Hz, ^3^*J*_HH_=5.4 Hz, 1H, H5’), 3.48-3.42 (dt, ^2^*J*_HH_=12.0 Hz, ^3^*J*_HH_=6.0 Hz, 1H, H5’’) ^13^C: 155.7 (C6), 153.0 (C2), 151.6 (C8), 150.2 (C4), 110.8 (C5), 86.1 (C1’), 85.1 (C4’), 70.5 (C2’), 70.5 (C3’), 62.0 (C5’), 56.5 (CH_3_O).

**8-methoxy-N^2^-[(dimethylamino)methylene]guanosine**

N,N-Dimethylformamide dimethyl acetal (1.6 ml, 12 mmol) was added to suspension of 8-methoxyguanosine (1.25 g, 4 mmol) in 15 ml of methanol. Reaction was heated at 60°C in an oil bath for 1.5h. The suspension was cooled down to approx -15°C and the precipitate was filtered, washed with acetone and dried over P_2_O_5_ in vacuo to yield 1.27 g (86%).

NMR (500 MHz, DMSO-d_6_) δ (ppm) ^1^H: 11.32 (brs, 1H, 1-NH), 8.44 (s, 1H, N^2^=CH), 5.66 (d, ^3^*J*_HH_=6.2 Hz, 1H, H1’), 5.30 (brs, 1H, 2’OH), 5.11 (brs, 1H, 3’OH), 4.84 (t, ^3^*J*_HH_=5.8, 6.1 Hz, 1H, 5’OH), 4.77 (t, ^3^*J*_HH_=4.9, 5.0 Hz, 1H, H2’), 4.11 (brs, 1H, H3’), 3.99 (s, 3H, 8-CH_3_O), 3.82 (q, ^3^*J*_HH_=4.8, 3.6, 1H, H4’), 3.59 (dt, ^2^*J*_HH_=11.9, ^3^*J*_HH_=4.9, 1H, H5’), 3.48 (dt, ^2^*J*_HH_=11.8 Hz, ^3^*J*_HH_=5.7 Hz, 1H, H5’’), 3.13, 3.01 (s, 6H, 2 x CH_3_) ^13^C: 157.7 (CH=N), 156.6 (C6), 156.4 (C2), 152.4 (C8), 148.8 (C4), 114.3 (C5), 86.3 (C1’), 85.2 (C4’), 70.9 (C2’), 70.5 (C3’), 62.0 (C5’), 56.6 (CH_3_O), 40.6, 34.6 (2 x CH_3_).

**5’-O-(4,4’-dimethoxytrityl)-8-methoxy-N^2^-[(dimethylamino)methylene]guanosine**

4,4′-Dimethoxytrityl chloride (2.25 mmol, 762 mg) was added to suspension of 8-methoxy-N^2^-[(dimethylamino)methylene]guanosine (1.5 mmol, 552 mg) in anhydrous pyridine (20 ml) and stirred at room temperature for 2h. After this time reaction was quenched with methanol. The solvents were evaporated, the operation was repeated twice with toluene. The product was purified on silica gel column chromatography with CH_2_Cl_2_:MeOH:Et_3_N (100:5:0.1 v/v/v) to yield 461 mg (46%).

NMR (500 MHz, DMSO-d_6_) δ (ppm) ^1^H: 11.32 (brs, 1H, 1-NH), 8.37 (s, 1H, N^2^=CH), 7.34 (d, 2H, DMT), 7.25-7.14 (m, 8H, DMT), 6.81 (t, ^3^*J*_HH_=9.4 Hz, 3H, DMT) 5.72 (d, ^3^*J*_HH_=4.6 Hz, 1H, H1’), 5.34 (d, ^3^*J*_HH_=5.6 Hz, 1H, 2’OH), 5.14 (d, , ^3^*J*_HH_=5.8 Hz, 1H, 3’OH), 4.70 (q, ^3^*J*_HH_=5.1 Hz, 1H, H2’), 4.27 (q, ^3^*J*_HH_=5.6, 5.5, 5.4 Hz, 1H, H3’), 3.94 (q, ^3^*J*_HH_=5.0, 4.8 Hz, 1H, H4’), 3.87 (s, 3H, 8-CH_3_O), 3.72 (s, 6H, 2x CH_3_O), 3.11 (d, ^3^*J*_HH_=4.6 Hz, 2H, H5’, H5”), 3.04, 3.00 (s, 6H, 2 x CH_3_) ^13^C: 158.0 (C-para, p-CH_3_OC_6_H_4_), 157.4 (CH=N), 156.6 (C6), 156.2 (C2), 152.3 (C8 ), 148.9 (C4), 144.9, 135.6, 135.5, 129.6, 129.5, 127.7, 127.6, 126.6 (DMT), 114.1 (C5), 113.0 (C, DMT) 86.6 (C1’), 85.2 (5’-C-O) 82.6 (C4’), 71.1 (C2’), 70.1 (C3’), 63.7 (C5’), 56.5 (CH_3_O), 55.0 (CH_3_O, DMT) 40.6, 34.6 (2 x CH_3_).

**2’-O-tert-butyldimethylsilyl-5’-O-(4,4’-dimethoxytrityl)-8-methoxy-N^2^-[(dimethylamino)methylene] guanosine**

Tert-butyldimethylsilyl chloride (1.47 mmol, 222 mg) was added to solution of imidazole (170 mg, 2.5 mmol) and 5’-O-DMT-mOG=dmf (656 mg, 0.98 mmol) in 10ml of DMF. The reaction was stirred for 50 h at room temperature, then the solvent was evaporated. Apart from 2’-O-TBDMS substituted product, two other products were obtained *i.e.* 3’-O-TBDMS and 2’,3’-di-O-TBDMS isomers. Any attempts to optimize reaction conditions did not gave us improvement of the yield. The product was purified on silica gel column chromatography with CH_2_Cl_2_:MeOH (100:2 v/v) to yield 205 mg (27%) of 2’-O-TBDMS isomer, 171 mg (19%) of 2’,3’-di-O-TBDMS isomer and 346 mg (45%) of 3’-O-TBDMS isomer.

NMR (500 MHz, DMSO-d_6_) δ (ppm) ^1^H: 11.34 (brs, 1H, 1-NH), 8.35 (s, 1H, N^2^=CH), 7.37 (d, 2H, DMT), 7.27-7.20 (m, 8H, DMT), 6.83 (t, ^3^*J*_HH_=8.7 Hz, 3H, DMT) 5.76 (d, ^3^*J*_HH_=6.0 Hz, 1H, H1’), 5.04 (d, ^3^*J*_HH_=6.0 Hz, 1H, 3’OH), 4.87 (t, ^3^*J*_HH_=5.3 Hz, 1H, H2’), 4.20 (q, ^3^*J*_HH_=5.1, 5.0 Hz, 1H, H3’), 3.98 (q, ^3^*J*_HH_=4.5, 4.7, 4.3 Hz 1H, H4’), 3.83 (s, 3H, 8-CH_3_O), 3.73 (s, 6H, 2x CH_3_O), 3.16 (dd, ^3^*J*_HH_=3.6, ^2^*J*_HH_=10.3 Hz, 1H, H5’), 3.11, (dd, ^3^*J*_HH_=5.5, ^2^*J*_HH_=10.1 Hz, 1H, H5”), 3.01, 3.00 (s, 6H, 2 x CH_3_), 0.77 (s, 9H, 3 x CH_3_), -0.02, -0.10 (s, 6H, (CH_3_)_2_Si). ^13^C: 158.0 (C-para, p-CH_3_OC_6_H_4_), 157.3 (CH=N), 156.6 (C6), 156.3 (C2), 152.3 (C8 ), 149.0 (C4), 144.9, 135.6, 135.4, 129.7, 129.6, 127.7, 127.6, 126.6 (DMT), 114.1 (C5), 113.1 (C, DMT), 85.2 (C1’, 5’-C-O), 83.0 (C4’), 72.6 (C2’), 70.0 (C3’), 63.4 (C5’), 56.4 (CH_3_O), 55.0 (2x CH_3_O, DMT) 40.5, 34.6 (2x CH_3_),25.5 (C(CH_3_)_3_), 17.9 (C(CH_3_)_3_), -4.86, -5.36 ((CH_3_)_2_Si).

**2’-O-tert-butyldimethylsilyl-3’-O-[(2-cyanoethoxy)-N,N,-diisopropylphosphoramidite]-5’-O-(4,4’-dimethoxytrityl)-8-methoxy-N^2^-[(dimethylamino)methylene]guanosine**

2-Cyanoethyl-N,N-diisopropylchlorophosphoramidite (55 ul, 0.247 mmol) was added to solution of tetrazole (13 mg, 0.18 mmol) and 2’-O-TBDMS-5’-O-DMT-mOG=dmf (140 mg, 0.18 mmol) in 1 ml of anhydrous acetonitrile and stirred for 2h at room temperature. The solvent was evaporated then product was purified on silica gel column chromatography with AcOEt:MeOH:Et_3_N (100:1:0.4 v/v/v). Fractions containing product were co-evaporated with benzene to obtain white foam. Yield 101 mg (57%).

NMR (500 MHz, DMSO-d_6_) δ (ppm) ^1^H: 11.35, 11.34 (brs, 2H, 1-NH), 8.34, 8.33 (s, 2H, N^2^=CH), 7.40-7.36 (dd, 4H, DMT), 7.29-7.21 (m, 16H, DMT), 6.86-6.82 (m, 6H, DMT), 5.81, 5.80 (d, ^3^*J*_HH_=6.5 Hz, 2H, H1’), 5.04, 4.92 (t, ^3^*J*_HH_=4.2-4.5, 5.4-5.6 Hz, 1H, H2’), 4.39-4.34 (m, 2H, H3’), 4.21, 4.12 (q, ^3^*J*_HH_=4.4, 4.2, 2.4 Hz 2H, H4’), 3.85-3.80 (s, 6H, 2x 8-CH_3_O, CH_2_O), 3.72 (brs, 12H, 4x CH_3_O), 3.36-3.55 (m, 6H, 4x CH of isopropyl, CH_2_O), 3.26-3.12 (m, 4H, 2x 5’-CH_2_) 3.01, 3.00 (s, 12H, 2 x CH_3_), 2.78-2.75 (m, 2H, CH_2_CN), 2.47-2.40 (m, 2H, CH_2_CN), 1.12, 0.99 (d, ^3^*J*_HH_=6.7 Hz, 24H, 8x CH_3_), 0.75, 0.74 (s, 18H, 6 x CH_3_),-0.03, -0.15, -0.17 ((s, 12H, 2 x (CH_3_)_2_Si) ^13^C: 158.1 (C-para, p-CH_3_OC_6_H_4_), 157.3 (CH=N), 156.6 (C6), 156.4 (C2), 152.1 (C8 ), 149.1 (C4), 144.9, 141.4, 140.1, 135.5, 135.3, 129.7, 129.6, 128.3, 127.8, 126.7 (DMT), 118.7 (CN), 114.1 (C5), 113.1 (C, DMT), 85.5, 85.4 (C1’),82.6, 82.3 (C4’, 5’-CO), 72.7 (C3’), 71.8 (C2’, C3’), 71.0 (C2’), 63.0 (C5’), 56.5 (CH_3_O), 56.4 (CH_2_O), 55.0 (2x CH_3_O, DMT), 42.3 (2x CH(CH_3_)_2_), 40.5, 34.5 (2 x CH_3_), 25.4 (C(CH_3_)_3_), 24.4, 24.2 (2x CH(CH_3_)_2_), 19.8 (CH_2_CN), 17.7 (C(CH_3_)_3_), -4.85, -5.52 ((CH_3_)_2_Si) ^31^P 149.3, 148.1

**8-benzyloxyguanosine**

Solution of 0.40 g of sodium dissolved in 15 ml of anhydrous benzyl alcohol was added to a solution of bromoguanosine (1.81 g, 5 mmol) in 30 ml of DMSO. The reaction mixture was heated at 75°C in an oil bath for 20h. The solution was cooled down to room temperature, neutralized with glacial acetic acid, diluted with acetone (50 ml) and poured into 250 ml of diethyl ether. The resulting precipitate was filtered, washed with acetone followed by cold water and then purified by crystallization from water and dried over P_2_O_5_ in vacuo to yield 1.16g (60%).

NMR (500 MHz, DMSO-d_6_) δ (ppm) ^1^H: 10.50 (brs, 1H, N1-H), 7.48-7.36 (m, 5H, C_6_H_5_), 6.33 (brs, 2H, NH_2_), 5.62 (d, ^3^*J*_HH_=6.2 Hz, 1H, H1’), 5.40 (s, 2H, OCH_2_), 5.33 (d, ^3^*J*_HH_=6.0 Hz, 1H, 2’OH), 5.00 (d, ^3^*J*_HH_=5.1 Hz, 1H, 3’OH), 4.85 (t, ^3^*J*_HH_=5.4, 5.8 Hz, 1H, 5’OH), 4.72 (q, ^3^*J*_HH_=5.6, 5.7, 5.9 Hz, 1H, H2’), 3.98 (q, ^3^*J*_HH_=4.7, 4.1, 4.8 Hz, 1H, H3’), 3.76 (q, ^3^*J*_HH_=5.2, 3.7, 5.3 Hz, 1H, H4’), 3.51-3.47 (1H, m, H5’), 3.41-3.36 (1H, m, H5’’)

**8-benzyloxy-N^2^-[(dimethylamino)methylene]guanosine**

N,N-Dimethylformamide dimethyl acetal (0.4 ml, 3 mmol) was added to suspension of 8-benzyloxyguanosine (0.59g, 1.5 mmol) in 6 ml of methanol. Reaction was heated at 60°C in an oil bath for 1 h. The suspension was cooled down to approx -15°C and the precipitate was filtered, washed with acetone and dried over P_2_O_5_ in vacuo to yield 0.6 g (91%).

NMR (500 MHz, DMSO-d_6_) δ (ppm) ^1^H: 11.34 (brs, 1H, 1-NH), 8.45 (s, 1H, N^2^=CH), 7.49 (d, ^3^*J*_HH_=7.1, 2H, H-*orto* of C_6_H_5_), 7.43-7.36 (m, 3H, H-*meta* H-*para* of C_6_H_5_), 5.69 (d, ^3^*J*_HH_=6.1 Hz, 1H, H1’), 5.44 (dd, ^2^*J*_HH_=12.3 Hz, 2H, CH_2_O), 5.30 (d, ^3^*J*_HH_=6.1 Hz, 1H, 2’OH), 5.08 (d, ^3^*J*_HH_=5.1 Hz, 1H, 3’OH), 4.82 (t, ^3^*J*_HH_=5.8, 6.2 Hz, 1H, 5’OH), 4.77 (q, ^3^*J*_HH_=5.9, 6.0 Hz, 1H, H2’), 4.05 (q, ^3^*J*_HH_=5.1, 4.0, 3.9 Hz, 1H, H3’), 3.80 (q, ^3^*J*_HH_=4.7, 4.1, 3.8, 1H, H4’), 3.53 (m, ^2^*J*_HH_=11.6, ^3^*J*_HH_=5.1, 1H, H5’), 3.43 (m, ^2^*J*_HH_=11.8, ^3^*J*_HH_=5.5, 1H, H5’’), 3.13, 3.02 (s, 6H, 2 x CH_3_) ^13^C: 157.6 (CH=N), 156.7 (C6), 156.5 (C2), 151.7 (C8), 148.8 (C4), 135.8, 128.5, 128.3, 128.1 (C_6_H_5_), 114.3 (C5), 86.4 (C1’), 85.1 (C4’), 70.9 (CH_2_O), 70.7 (C2’), 70.4 (C3’), 62.0 (C5’), 40.6, 34.6 (2 x CH_3_).

**8-benzyloxy-5’-O-(4,4’-dimethoxytrityl)-N^2^-[(dimethylamino)methylene]guanosine**

4,4′-Dimethoxytrityl chloride (2.0 mmol, 676 mg) was added to suspension of 8-benzyloxy-N^2^-[(dimethylamino)methylene]guanosine (1.66 mmol, 715 mg) in anhydrous pyridine (15 ml) and stirred at -15°C for 1.5 h. After this time reaction was quenched with methanol. The solvents were evaporated, the operation was repeated twice with toluene. The product was purified on silica gel column chromatography with CH_2_Cl_2_:MeOH:Et_3_N (100:5:0.1 v/v/v) to yield 502 mg (41%).

NMR (500 MHz, DMSO-d_6_) δ (ppm) ^1^H: 11.34 (brs, 1H, 1-NH), 8.40 (s, 1H, N^2^=CH), 7.36-7.29 (m, 7H, C_6_H_5_ or C_6_H_4_), 7.24-7.15 (m,7H, C_6_H_5_ or C_6_H_4_), 6.79 (t, ^3^*J*_HH_=8.8 Hz, 4H, C_6_H_4_) 5.78 (d, ^3^*J*_HH_=4.1 Hz, 1H, H1’), 5.38 (d, ^2^*J*_HH_=12.1 Hz, 1H, CH_2_O), 5.37 (d, 1H, 2’OH), 5.27 (d, ^2^*J*_HH_=12.1 Hz, 1H, CH_2_O), 5.07 (d, ^3^*J*_HH_=6.3 Hz, 1H, 3’OH), 4.66 (q, ^3^*J*_HH_=4.0, 4.5, 5.7 Hz, 1H, H2’), 4.10 (q, ^3^*J*_HH_=5.4, 5.6, 4.8 Hz, 1H, H3’), 3.93-3.89 (m, 1H, H4’), 3.71 (s, 6H, 2x CH_3_O), 3.11-3.06 (dd, ^2^*J*_HH_=10.0, ^3^*J*_HH_=5.1, 1H, H5’), 3.03-3.00 (m,1H, H5’’), 3.05, 3.00 (s, 6H, 2 x CH_3_) ^13^C: 158.0 (C-para, p-CH_3_OC_6_H_4_), 157.5 (CH=N), 156.7 (C6), 156.3 (C2),151.5 (C8), 148.7 (C4),144.8, 135.6, 135.5, 129.6, 128.4, 128.3, 127.9, 127.6, 126.6 (5’-O-DMT, 8-C_6_H_5_CH_2_O), 114.1 (C5), 113.0 (C, DMT), 87.0 (C1’), 85.2 (5’-C-O), 82.5 (C4’), 71.4 (C2’), 70.7 (CH_2_O), 70.3 (C3’), 64.1 (C5’), 55.0 (2x CH_3_O, DMT), 40.6, 34.6 (2 x CH_3_).

**8-benzyloxy-2’-O-tert-butyldimethylsilyl-5’-O-(4,4’-dimethoxytrityl)-N^2^-[(dimethylamino)methylene] guanosine**

Tert-butyldimethylsilyl chloride (0.67 mmol, 101 mg) was added to solution of imidazole (83 mg, 1.2 mmol) and 5’-O-DMT-bnOG=dmf (448 mg, 0.61 mmol) in 7 ml of DMF. The reaction was stirred for 48h at room temperature, then the solvent was evaporated. Apart from 2’-O-TBDMS substituted product, two other products were obtained i.e. 3’-O-TBDMS and 2’,3’-di-O-TBDMS isomers. Any attempts to optimize reaction conditions did not gave us improvement of the yield. The product was purified on silica gel column chromatography with AcOEt:MeOH:Et_3_N (100:2:0.1 v/v/v) to yield 136 mg (26%) of 2’-O-TBDMS isomer, 27 mg (4%) of 2’,3’-di-O-TBDMS isomer and 183 mg (35%) of 3’-O-TBDMS isomer.

NMR (500 MHz, DMSO-d_6_) δ (ppm) ^1^H: 11.36 (brs, 1H, 1-NH), 8.33 (s, 1H, N^2^=CH), ), 7.35-7.17 (m, 14H, C_6_H_5_ or C_6_H_4_), 6.80 (t, ^3^*J*_HH_=9.2 Hz, 4H, C_6_H_4_), 5.78 (d, ^3^*J*_HH_=4.3 Hz, 1H, H1’), 5.37 (d, ^2^*J*_HH_=12.1 Hz, 1H, CH_2_O), 5.30 (d, ^2^*J*_HH_=12.1 Hz, 1H, CH_2_O), 4.97 (d, ^3^*J*_HH_=6.6 Hz, 1H, 3’OH), 4.75 (brs, 1H, H2’), 4.09 (q, ^3^*J*_HH_=5.4, 5.5, 5.0 Hz, 1H, H3’), 3.95 (q, ^3^*J*_HH_=5.8, 3.9, 6.3 Hz, 1H, H4’), 3.71 (s, 6H, 2x CH_3_O), 3.14 (dd, ^2^*J*_HH_=10.0, ^3^*J*_HH_=6.3, 1H, H5’), 3.05 (dd, ^2^*J*_HH_=10.0, ^3^*J*_HH_=4.1, 1H, H5’’), 3.00, 2.89 (s, 6H, 2 x CH_3_), 0.74 (s, 9H, 3 x CH_3_), -0.07, -0.15 (s, 6H, (CH_3_)_2_Si). ^13^C: 158.0 (C-para, p-CH_3_OC_6_H_4_), 157.3 (CH=N), 156.7 (C6), 156.3 (C2),151.4 (C8), 148.7 (C4),144.9, 135.5, 135.4, 129.6, 128.4, 128.3, 128.0, 127.7, 126.6 (5’-O-DMT, 8-C_6_H_5_CH_2_O), 114.1 (C5), 113.0 (C, DMT), 86.8 (C1’), 85.2 (5’-C-O), 82.7 (C4’), 73.2 (C2’), 70.7 (CH_2_O), 70.2 (C3’), 63.8 (C5’), 55.0 (2x CH_3_O, DMT), 40.6, 34.6 (2 x CH_3_), ,25.5 (C(CH_3_)_3_), 17.9 (C(CH_3_)_3_), -4.82, -5.40 ((CH_3_)_2_Si).

**8-benzyloxy-2’-O-tert-butyldimethylsilyl-3’-O-[(2-cyanoethoxy)-N,N,-diisopropylphosphoramidite]-5’-O-(4,4’-dimethoxytrityl)-N^2^-[(dimethylamino)methylene] guanosine**

2-Cyanoethyl-N,N-diisopropylchlorophosphoramidite (45 µl, 0.2 mmol) was added to solution of tetrazole (12 mg, 0.17 mmol) and 2’-O-TBDMS-5’-O-DMT-bnOG=dmf (134 mg, 0.16 mmol) in 1 ml of anhydrous acetonitrile and stirred for 2h at room temperature. The solvent was evaporated then product was purified on silica gel column chromatography with AcOEt:MeOH:Et_3_N (100:1:0.4 v/v/v). Fractions containing product were co-evaporated with benzene to obtain white foam. Yield 94 mg (55%).

NMR (500 MHz, DMSO-d_6_) δ (ppm) ^1^H: 11.36 (brs, 2H, 1-NH), 8.29, 8.23 (s, 2H, N^2^=CH), 7.35-7.16 (m, 26H, DMT), 6.81-6.77 (m, 10H, DMT), 5.81, 5.80 (d, 2H, H1’), 5.37, 5.33 (brd, 4H, CH_2_O), 5.01, 4.79 (brs, 2H, H2’), 4.28 (brs, 1H, H3’), 4.06 (m, 1H, H3’), 4.03-3.98 (m, 2H, H4’), 3.85-3.80 (s, 6H, 2x 8-CH_3_O, CH_2_O), 3.72 (brs, 12H, 4x CH_3_O), 3.36-3.55 (m, 6H, 4x CH of isopropyl, CH_2_O), 3.26-3.12 (m, 4H, 2x 5’-CH_2_) 3.01, 3.00 (s, 12H, 2 x CH_3_), 2.78-2.75 (m, 2H, CH_2_CN), 2.47-2.40 (m, 2H, CH_2_CN), 1.12, 0.99 (d, ^3^*J*_HH_=6.7 Hz, 24H, 8x CH_3_), 0.75, 0.74 (s, 18H, 6 x CH_3_),-0.03, -0.15, -0.17 ((s, 12H, 2 x (CH_3_)_2_Si) ^31^P 149.3, 148.4. ^13^C: 158.0 (C-para, p-CH_3_OC_6_H_4_), 157.3 (CH=N), 156.7 (C6), 156.3 (C2),151.4 (C8), 148.7 (C4),144.9, 135.5, 135.4, 129.6, 128.4, 128.3, 128.0, 127.7, 126.6 (5’-O-DMT, 8-C_6_H_5_CH_2_O), 118.5 (CN), 114.1 (C5), 113.0 (C, DMT), 86.8 (C1’), 85.2 (5’-C-O), 82.7 (C4’), 73.4 (C2’), 70.5 (CH_2_O), 70.2 (C3’), 63.8 (C5’), 56.4 (CH_2_O), 55.0 (2x CH_3_O, DMT), 42.5 (2x CH(CH_3_)_2_), 40.7, 34.5 (2 x CH_3_), ,25.3 (C(CH_3_)_3_), 24.5, 24.3 (2x CH(CH_3_)_2_), 19.8 (CH_2_CN) 17.8 (C(CH_3_)_3_), -4.80, -5.50 ((CH_3_)_2_Si) ^31^P 149.3, 148.4.
