# Supplementary material for: Hybridization Properties of RNA Containing 8-Methoxyguanosine and 8-Benzyloxyguanosine
Source: PLoS One. 2015 Sep 9;10(9):e0137674. doi: 10.1371/journal.pone.0137674 (PMC4564172; doi:10.1371/journal.pone.0137674)
Supplement: S1 Table — (DOCX) [file pone.0137674.s003.docx]

**S1 Table. Thermodynamic parameters of duplex formation.**^a^

| Duplexes (5'-3') | | Average of curve fits | | | |  | T_M_^-1^ vs log C_T_ plots | | | | | | | |
| --- | --- | --- | --- | --- | --- | --- | --- | --- | --- | --- | --- | --- | --- | --- |
|  |  | **-ΔH˚ (kcal/mol)** | **-ΔS˚ (eu)** | **-ΔG˚_37_ (kcal/mol)** | **T_M_ ^b^ (˚C)** |  | **-ΔH˚ (kcal/mol)** | **-ΔS˚ (eu)** | **-ΔG˚_37_ (kcal/mol)** | **T_M_^b^ (˚C)** | **ΔΔG˚_37_ (kcal/mol)** | **ΔT_M_^b^ (˚C)** | **ΔΔG˚_37_ (kcal/mol)** | **ΔT_M_^b^ (˚C)** |
| GAUCGACAG | CUGUCGAUC | 81.8±0.9 | 228.2±2.6 | 11.06±0.04 | 55.2 |  | 80.0±1.3 | 222.4±4.1 | 10.98±0.05 | 55.3 | 0 | 0 | - | - |
| GAUC**G^Bx^**ACAG | CUGUCGAUC | (43.4±2.5) | (116.4±7.6) | (7.32±0.53) | (42.7) |  | (100.1±5.4) | (299.4±17.4) | (7.29±0.05) | (39.3) | 3.69 | -16.0 | **0** | **0** |
| GAUC**G^Mx^**ACAG | CUGUCGAUC | 76.3±1.5 | 216.3±5.0 | 9.18±0.06 | 48.2 |  | 72.7±2.8 | 204.9±8.7 | 9.11±0.07 | 48.4 | 1.87 | -6.9 | *0* | *0* |
| GAUCGACAG | CUGUAGAUC | 55.±1.6 | 158.4±5.2 | 6.08±0.06 | 34.5 |  | 57.7±4.3 | 166.7±14.2 | 6.03±0.12 | 34.3 | 4.95 | -21.0 | - | - |
| GAUC**G^Bx^**ACAG | CUGUAGAUC | (47.5±1.0) | (127.8±3.5) | (7.83±0.23) | (47.7) |  | (75.5±4.2) | (217.6±13.4) | (7.99±0.05) | (43.1) | - | - | **-0.70** | **3.8** |
| GAUC**G^Mx^**ACAG | CUGUAGAUC | 54.0±12.2 | 155.7±39.6 | 5.76±0.24 | 32.6 |  | 53.4±3.4 | 153.6±11.2 | 5.71±0.13 | 32.3 | - | - | *3.40* | *-16.1* |
| GAUCGACAG | CUGUGGAUC | 69.8±1.0 | 203.2±3.4 | 6.75±0.10 | 38.0 |  | 66.0±2.5 | 190.7±8.2 | 6.80±0.04 | 38.3 | 4.18 | -17.0 |  |  |
| GAUC**G^Bx^**ACAG | CUGUGGAUC | (44.4±2.8) | (121.4±8.3) | (6.78±0.57) | (38.8) |  | (146.0±7.7) | (452.6±25.1) | (5.66±0.10) | (35.2) | - | - | **1.63** | **-4.1** |
| GAUC**G^Mx^**ACAG | CUGUGGAUC | 79.3±0.8 | 230.8±2.6 | 7.66±0.05 | 41.5 |  | 79.3±1.5 | 230.8±4.7 | 7.67±0.01 | 41.5 | - | - | *1.44* | *-6.9* |
| GAUCGACAG | CUGUUGAUC | 78.2±0.7 | 226.8±2.1 | 7.91±0.05 | 42.6 |  | 77.1±1.5 | 222.9±4.8 | 7.91±0.01 | 42.6 | 3.07 | -12.7 | - | - |
| GAUC**G^Bx^**ACAG | CUGUUGAUC | (64.4±11.1) | (188.8±35.9) | (5.81±0.03) | (33.6) |  | (102.6±6.1) | (315.8±20.2) | (4.68±0.20) | (31.5) | - | - | **2.61** | **-7.8** |
| GAUC**G^Mx^**ACAG | CUGUUGAUC | 72.7±1.8 | 213.6±5.8 | 6.42±0.06 | 36.5 |  | 75.6±1.2 | 223.1±4.0 | 6.37±0.02 | 36.4 | - | - | *2.74* | *-12.0* |

**G^Mx^** – 8-methoxyguanosine, **G^Bx^** – 8-bezyloxyguanosine

a – 100 mM NaCl, 20mM sodium cacodylate, 0.5 mM Na_2_EDTA, pH 7.0, b - calculated for 10^-4^ M oligomer concentration
